# Applications and Mechanisms of Stimuli-Responsive Hydrogels in Traumatic Brain Injury

**DOI:** 10.3390/gels8080482

**Published:** 2022-08-01

**Authors:** Xingfan Li, Linyan Duan, Mingyue Kong, Xuejun Wen, Fangxia Guan, Shanshan Ma

**Affiliations:** 1 School of Life Sciences, Zhengzhou University, Zhengzhou 450001, China; lixingfan0929@163.com (X.L.); duanlinyan4881@163.com (L.D.); 2 NHC Key Laboratory of Birth Defects Prevention, Henan Key Laboratory of Population Defects Prevention, Zhengzhou 450002, China; mykong2021@163.com; 3 Department of Chemical and Life Science Engineering, School of Engineering, Virginia Commonwealth University, Richmond, VA 23284, USA; xwen@vcu.edu

**Keywords:** stimuli-responsive hydrogels, traumatic brain injury, biomedical applications, therapeutic mechanisms

## Abstract

Traumatic brain injury (TBI) is a global neurotrauma with high morbidity and mortality that seriously threatens the life quality of patients and causes heavy burdens to families, healthcare institutions, and society. Neuroinflammation and oxidative stress can further aggravate neuronal cell death, hinder functional recovery, and lead to secondary brain injury. In addition, the blood–brain barrier prevents drugs from entering the brain tissue, which is not conducive to the recovery of TBI. Due to their high water content, biodegradability, and similarity to the natural extracellular matrix (ECM), hydrogels are widely used for the delivery and release of various therapeutic agents (drugs, natural extracts, and cells, etc.) that exhibit beneficial therapeutic efficacy in tissue repair, such as TBI. Stimuli-responsive hydrogels can undergo reversible or irreversible changes in properties, structures, and functions in response to internal/external stimuli or physiological/pathological environmental stimuli, and further improve the therapeutic effects on diseases. In this paper, we reviewed the common types of stimuli-responsive hydrogels and their applications in TBI, and further analyzed the therapeutic effects of hydrogels in TBI, such as pro-neurogenesis, anti-inflammatory, anti-apoptosis, anti-oxidation, and pro-angiogenesis. Our study may provide strategies for the treatment of TBI by using stimuli-responsive hydrogels.

## 1. Introduction

Traumatic brain injury (TBI) is a major public health problem with an increasing global prevalence and high mortality that seriously threatens the life quality of patients and causes heavy burdens to families, healthcare institutions, and society [1,2]. The global incidence of TBI is about 870–1000 cases per 100,000 people, and an estimated 64–74 million individuals worldwide suffer from TBI every year [3]. According to the Glasgow Coma Score (GCS), TBI can be divided into mild injury, moderate injury, and severe injury [4]. The pathological process of TBI includes primary injury and secondary injury. Primary brain injury generally refers to the direct damage to nerve tissue caused by external forces such as skull deformation, intracranial hemorrhage, brain tissue contusion, axonal injury, etc. [5]. Secondary brain injury occurs several minutes after injury and lasts for a long time, and includes the disturbance of ion homeostasis, excitatory toxicity, oxidative stress, inflammation, and cell death [6]. Compared with primary brain injury, secondary brain injury brings more serious harm [7]. Over the past few decades, the therapeutic outcomes for TBI patients have substantially improved due to the increasing number of basic, nursing, and clinical studies related to TBI [8]. However, due to the existence of the blood–brain barrier (BBB), most compounds or drugs cannot effectively cross the BBB to reach the injury site, thereby affecting their efficacy [9]. Therefore, there is still no effective treatment for TBI till now. Research has focused on alleviating secondary brain injury, enhancing neural network reorganization, and promoting functional recovery.

The boom in biomaterials has provided new techniques and strategies to help overcome the BBB [10]. Hydrogels are widely used for the delivery and release of various therapeutic agents (drugs, natural extracts, and cells, etc.) due to their high water content, biodegradability, and similarity to the natural extracellular matrix (ECM); these exert beneficial therapeutic efficacy in many diseases, such as TBI [11]. In addition to these features, injectable hydrogels also have special advantages, such as minimal invasiveness, effective defect filling, etc. [12]. Due to the variability and adjustability of the microenvironment, hydrogels need to be responsive to biophysical and biochemical signals, which is beneficial for function repair. Therefore, researchers have developed stimuli-responsive hydrogels [13].

Stimuli-responsive hydrogels can undergo reversible or irreversible changes in properties, structures, and functions in response to internal/external stimuli (e.g., magnetic fields, light, ultrasound, mechanical force) or physiological/pathological environmental stimuli (e.g., enzymatic reactions, temperature), and further gain some specific effects (e.g., sustained release, transport, alignment) [14,15]. Furthermore, stimuli-responsive hydrogels can rapidly detect and respond to stimuli without damaging normal cells and tissues, thereby greatly improving the therapeutic efficacy and reducing side effects [16,17]. Several articles have discussed the use of bioactive matrices (stem cells, hydrogels, and their combined systems) in central nervous system diseases, such as TBI, but they focused on hydrogel-related stem cell therapy or bioactive matrices in neural repair [10,11]. In this paper, we reviewed the characteristics, synthesis mechanisms of stimuli-responsive hydrogels, and further analyzed the applications and different therapeutic effects of hydrogels in TBI, such as anti-inflammatory, anti-oxidation, anti-apoptosis, pro-neurogenesis, and pro-angiogenesis. Our study may provide strategies for the recovery of TBI by using stimuli-responsive hydrogels, which may facilitate the synthesis of more intelligent biomaterials in the future to satisfy applications in TBI.

## 2. Stimuli-Responsive Hydrogels

In the past decades, stimuli-responsive hydrogels have received increasing attention as “smart hydrogels” with various advantages and functions [15,17,18]. Here, we summarized the common stimuli-responsive hydrogels, and described their synthetic materials, gelation mechanisms, and properties.

### 2.1. Thermo-Responsive Hydrogels

Thermo-responsive hydrogels, one of the most widely studied stimuli-responsive hydrogels, usually contain different hydrophobic groups (such as methyl, ethyl, and propyl groups), which can change their physical and conformational properties with the change of temperature [19,20]. Biomaterials used in the synthesis of thermo-responsive hydrogels can be divided into natural polymers and synthetic polymers. Natural polymers include polysaccharides (cellulose, chitosan, and xyloglucan, etc.), proteins (gelatin), and so on. Synthetic polymers include poly(*N*-isopropylacrylamide) (pNIPAAm), poly (ethylene oxide)-poly (propylene oxide)-poly (ethylene oxide) copolymer (PEO-PPO-PEO), polyethylene glycol ester (PEG)/biodegradable polyester, poly (organophosphoronitrile), and dimethylamine (ethyl methacrylate), etc. [21].

**Figure 1:**
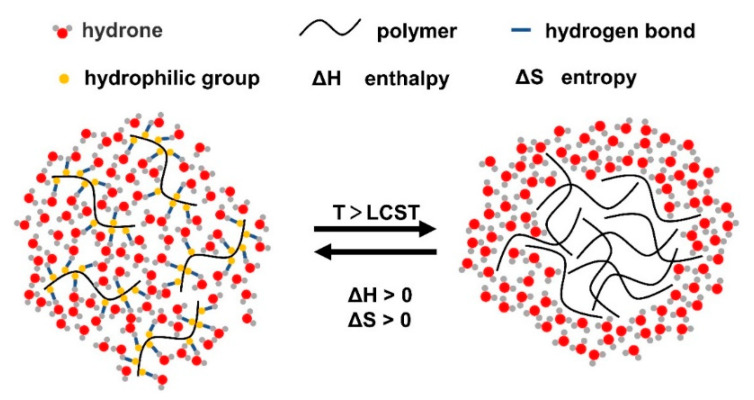
Schematic of thermoresponsive hydrogels (LCST hydrogels). With the increase in temperature, the hydrogen bond force between polymers and water molecules is weakened, and the hydrophobic effect is enhanced, forming the hydrogel.

The gelling principle of thermoresponsive hydrogels is that when the temperature is lower than the lower critical solution temperature (LCST), the hydrophilic groups in the system form hydrogen bonds with hydrophilic molecules in the environment, which increases the solubility of hydrogels and can be in situ injected in the form of a solution. With the increase in temperature, the hydrogen bond force gradually weakens, while the interaction between hydrophobic groups in the system gradually strengthens, and the solubility decreases (Figure 1). After reaching the LCST, a gel is formed [20,22]. Therefore, thermoresponsive hydrogels can be divided into two systems with LCST and upper critical solution temperature (UCST) according to different properties. For LCST hydrogels, the solubility is nonlinear and inversely proportional to temperature [23]. When the ambient temperature is above the LCST, the system is transformed from a solution to a gel. Due to this mechanism, the hydrogel solution becomes liquid at room temperature and becomes a gel at physiological temperature, making the hydrogel injectable [24]. UCST hydrogels have the opposite properties to LCST hydrogels. When the ambient temperature is below the UCST, the system changes from a solution to a gel. As such hydrogels often require high-temperature injection, which may lead to denaturation of the loaded drugs or proteins, they are not widely used in the biomedical field [25].

Thermoresponsive hydrogels are widely used in drug release and cell encapsulation [26]. Pan et al. loaded black phosphorus nanosheets (BPNs) on a platelet-rich plasma (PRP)-chitosan thermoresponsive hydrogel. With an increase in temperature, the proton transfer of the amino group on the chitosan molecule reduced the electrostatic repulsion between chitosan molecules and increased the aggregation of hydrophobic groups, thus forming the hydrogel. Under the action of a photothermal effect, this thermoresponsive hydrogel controlled the slow release of BPNs degradation products, which is beneficial to the treatment of rheumatoid arthritis (RA) [27]. In addition, the poly[(propylenesulfide)-block-(*N*,*N*-dimethylacrylamide)-block-(*N*-isopropylacrylamide)] (PPS-b-PDMA-b-PNIPAAM) hydrogel was a thermoresponsive hydrogel. At 25 °C, it assembled into micelles that included a hydrophobic PPS core and PNIPAAM on the outer corona. When the temperature exceeded the LCST of PNIPAAM, the micelles sharply transitioned into stable, hydrated gels that demonstrated ROS-dependent drug release [28]. Collectively, thermoresponsive hydrogels are particularly promising for future applications in drug delivery. However, the translation and application of this hydrogel at the clinical level still require more in-depth research to improve their biocompatibility, safety, and experimental reproducibility.

### 2.2. Photoresponsive Hydrogels

Generally speaking, photoresponsive hydrogels mainly respond to ultraviolet (UV), visible, and near infrared (NIR) light [29]. Photoresponsive hydrogels are composed of polymer networks including photosensitive moieties with reversible crosslinking and photothermal capability [30]. After being stimulated by light, the photosensitive part captures the light signal and converts it into a chemical signals through the reactions of isomerization, cleavage and dimerization, causing changes in the structure or properties, such as shrinking swelling or crosslinking [31].

**Figure 2:**
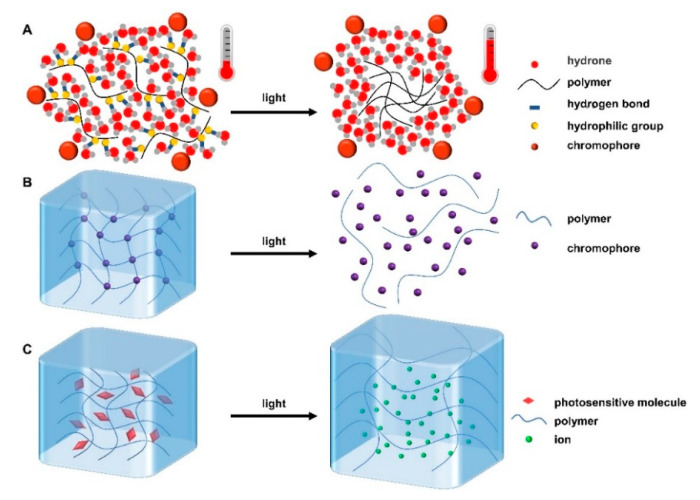
Three gelation mechanisms of photoresponsive hydrogels. When stimulated by light, (**A**) chromophore converts light energy into heat energy, encouraging polymers to aggregate into gels. (**B**) The chromophore structure and properties in the micelles change, causing the hydrogel to become uncrosslinked. (**C**) Photosensitive molecules dissociate, resulting in internal and external osmotic pressure, and causing hydrogel expansion.

There are three gelation mechanisms for photoresponsive hydrogels. (i) The chromophores groups are added into the whole hydrogel system. These chromophores have the ability to convert light energy into heat energy, so that the hydrogen bond force in the system will weaken and then the structure or properties of the hydrogels will change; this is similar to thermoresponsive hydrogels (Figure 2A) [32]. (ii) The chromophore groups incorporated into the hydrogel matrix undergo isomerization, photocleavage, or photooxidation after photo/light stimulation, leading to changes in their physical or chemical properties, thus affecting the structure of hydrogels and even causing the hydrogels to become uncrosslinked (Figure 2B) [33]. (iii) The photosensitive molecules dissociate after being stimulated by light, resulting in a large number of ions. The ionic concentration gradient generated inside and outside the gel can enhance the osmotic pressure, causing the gel to swell (Figure 2C) [34]. For example, Wang et al. prepared a polydopamine/collagen/silk fibroin photoresponsive hydrogel. Polydopamine, which could be cross-linked to collagen or silk fibroin through catechin groups, accelerated hydrogel formation and imparted superior photothermal properties to the hydrogel. Under the irradiation of NIR light, it released thrombin, thus promoting blood coagulation, preventing angiogenesis, and effectively inhibiting the recurrence and metastasis of triple-negative breast cancer [35]. In addition, Wang et al. prepared an injectable redox and light-responsive bio-inspired MnO_2_ hybrid (BMH) hydrogel through non-covalent self-assembly and MnO_2_ nanosheets mediated the covalent oxidative polymerization of the catechol-functionalized chitosan. Under the action of NIR, this light-responsive BMH hydrogel inhibited melanoma growth and had long-term antibacterial properties through the controllable release of DOX and photothermal therapy [36].

The advantage of photoresponsive hydrogels is that the photo/light-energy stimulation is controllable and drug delivery can be precisely targeted in time and space [31]. However, there are also certain limitations. First, UV is a high-energy electromagnetic wave with carcinogenic potential and poor tissue penetration, which makes it difficult to treat deeply injured sites. The current solution is to incorporate upconverting nanoparticles (UCNPs), which can convert NIR energy into UV energy [37]. Besides, as the photosensitive groups necessary for photoresponsive hydrogels may be toxic, better biocompatible materials need to be further investigated in the future [38].

### 2.3. Magnetic-Responsive Hydrogels

Magnetic-responsive hydrogels are synthesized by incorporating magnetic nanoparticles into crosslinked polymers; these particles can change their structures, properties, and functions in response to magnetic fields [39]. In addition to Fe_3_O_4_, other common magnetic nanoparticles include Fe_3_O_4_@SiO_2_, Zn_0.47_Mn_0.53_Fe_2_O_4_, γ-Fe_2_O_3_, and Co_3_O_4_ [40]. There are three common synthesis methods for magnetic-responsive hydrogels: co-precipitation or embedding, grafting, and blending [41].

**Figure 3:**
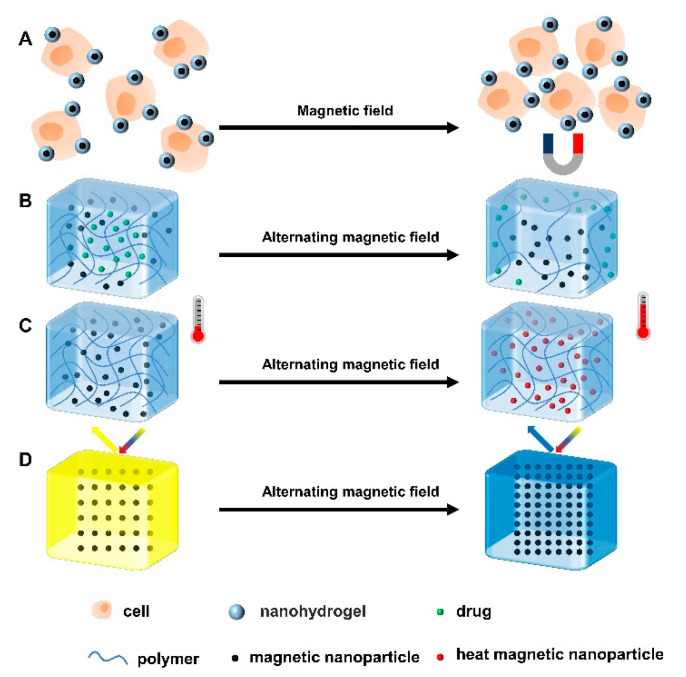
Four gelation mechanisms of magnetic-responsive hydrogels. (**A**) Under the influence of a magnetic field, the cells attached to the magnetic nanoparticle hydrogel clustered in a specific direction. Under the action of alternating magnetic field, (**B**) magnetic nanoparticles in hydrogels move, resulting in a decrease in crosslinking density and the release of loaded drugs. (**C**) The motion of magnetic nanoparticles generates heat, which leads to a temperature rise in the system. (**D**) Due to the magnetothermal effect, the distance between the magnetic nanoparticles decreases and the diffraction peak shifts, leading to the discoloration of the hydrogel.

Under the stimulation of magnetic field, the magnetic nanoparticles in the nanohydrogel can self-assemble to form an ordered structure, making the surface of the hydrogel anisotropic (Figure 3A) and thereby promoting cell proliferation, neurogenesis, signal transduction and ECM regeneration [42]. Besides, the macroscopic magnetic-responsive hydrogels perform functions such as magnetron release, magnetocaloric effect, and optical property adjustment under magnetic field stimulation (Figure 3B) [43]. Magnetic actuation does not require contact stimulation and can be operated remotely, which is suitable for complex environments [44,45]. Under the stimulation of an alternating magnetic field (AMF), the magnetic nanoparticles vibrate and the temperature increases, which accelerates the movement of drug molecules and the degradation of polymers, thus promoting drug release (Figure 3C). In addition, it is also used in combination with chemotherapy to treat tumors [46]. However, magnetic nanoparticles tend to aggregate under the action of a magnetic field, resulting in reduced pores between nanoparticles and, in turn, limiting the release of drugs [47]. In addition, under the action of a magnetic field, the magnetic-responsive hydrogel undergoes a magnetocaloric effect, which may lead to a shift in the diffraction peaks and thus the discoloration of the hydrogel (Figure 3D) [48]. For example, Zhang et al. prepared hydrogels using hyaluronic acid (HA) and PEG, and then added magnetic nanoparticles and type II collagen to the gel. This hydrogel could respond to an external magnetic field and travel to the tissue defect sites in physiological fluids under remote magnetic guidance [49]. To sum up, magnetic-responsive hydrogels have the advantages of low invasiveness, good tissue permeability, remote operation, rapid response and high controllability, but they also have some limitations, such as low mechanical strength, material toxicity, and low biocompatibility [41,43].

### 2.4. Electroresponsive Hydrogels

Electroresponsive hydrogels are synthesized by adding electroactive materials into hydrogel matrix. Common electroactive materials are divided into two categories: natural materials (such as metals) and inorganic vs. organic conductive materials [such as graphene, carbon nanotubes, polypyrrole (PPy), polyaniline (PANI), and poly3,4-ethylenedioxythiophene (PEDOT)] [50,51]. Electroresponsive hydrogels can respond to electric field stimulation to modify their structures and properties and perform specific functions.

Under the stimulation of an electric field, the charges in the gel solution move directionally, and the electroactive materials undergo electrochemical reactions, resulting in structural changes to the hydrogel, thereby achieving the purpose of regulating drugs release [52,53]. In addition, electroresponsive hydrogels can promote signal transduction, thereby affecting cell growth and proliferation [54]. Moreover, electroresponsive hydrogels can also affect cellular processes, such as angiogenesis, neurogenesis, or cardiogenesis under electric field stimulation, showing good potential in biomedical engineering [52,55]. For instance, chitosan can be electro-activated by aniline–pentamer segments, and an injectable electroactive hydrogel based on pluronic–chitosan/aniline–pentamer clearly improved hippocampal-dependent learning and memory performance [56]. However, the biocompatibility, degradability, and stability of electroresponsive hydrogels still need to be improved [51].

### 2.5. Bioresponsive Hydrogels

Bioresponsive hydrogels can change their structures or properties (e.g., swelling, contraction, dissociation) in the presence of specific biological factors or activity and concentration changes [57]. Bioresponsive hydrogels are mainly divided into two categories: hydrogels coated with specific enzyme reaction substrates [58] and hydrogels coated with specific antigens or antibodies [59].

Enzyme-responsive hydrogels are highly selective, using a specific substrate as a crosslinking agent. In the presence of specific enzymes, the substrate undergoes enzymatic hydrolysis resulting in the cross-linking of hydrogels to change their structures or properties [22]. Enzyme-responsive hydrogels system must satisfy three conditions. First, the polymer network must contain specific enzyme substrates. Second, these substrates should be close to the active center of the enzyme. Third, the enzymatic reactions of these substrates can cause changes in the structure or properties of hydrogels [60]. Shen et al. used the polymer PLGA-PEI-MPEG (PPP) as a carrier and loaded it with activated cell-penetrating peptides (ACPP) and etanercept (ET) to construct an ET@PPP-ACPP hydrogel. ACPP and matrix metalloproteinases (MMPs) have affinities that can give hydrogels the ability to target lesions and improve the therapeutic effect in spinal cord injury (SCI) [61]. In addition, Katayama et al. synthesized polymer–peptide conjugates, NIPAM−PEP and NIPAM−PEPEP, which are graft-type copolymers, each synthesized by a methacryloyl monomer with radical copolymerization. The polymer was coated with a substrate peptide of protein kinase A (PKA) in response to PKA phosphorylation. Subsequently, the LCST of the polymer was elevated and decomposed under activation by PKA, releasing the drugs into it [62].

**Figure 4:**
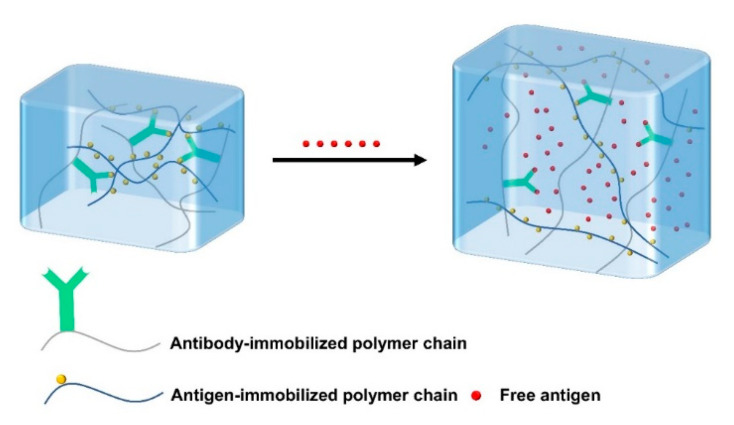
Schematic of the antigen-responsive hydrogels. The free antigens in the environment compete with the binding antigens in the hydrogel, resulting in the decrease of crosslinking density and swelling of the hydrogel.

Antigen-responsive hydrogels covalently bind antigens and antibodies into hydrogels. Due to the non-covalent binding between antigen and antibody, the antigen-responsive hydrogels are reversibly crosslinked. The free antigens presented in the environment can compete with the antigens in the hydrogel (Figure 4), leading to the weakness of the interaction between the antigen and the antibody in the hydrogel, thereby reducing the crosslinking density of the hydrogel and causing swelling or osmosis [63]. Ye et al. prepared a bioresponsive hydrogel using antigen–antibody binding sites as crosslinking points to prepare IgG sensing gratings, which can detect human immunoglobulin G (H-IgG) in solution with high specificity and sensitivity [64]. In short, bioresponsive hydrogels generally respond to a specific substance (such as enzymes, antigens, antibodies, etc.) with high biocompatibility, and have good application prospects in the biomedical field.

## 3. Applications of Stimuli-Responsive Hydrogels in TBI

TBI-mediated chronic inflammation and oxidative stress in the microenvironment further induce neuronal death and lead to persistent neuronal damage, which often cause motor dysfunction, speech impairment, and intellectual disability [65,66]. Traditional drug delivery is hampered by the presence of the BBB, making it difficult to reach the target area [67]. Hydrogels have good biocompatibility and injectability, which allows them to injected in situ and adapt to the irregularity of the injury site, and have a wide range of applications in TBI [68]. Stimuli-responsive hydrogels can respond to the inflammatory and oxidative stress microenvironment at the injury site to regulate inflammation, edema, and oxidative stress levels, and promote neurogenesis and functional recovery [11].

Yao et al. synthesized a thermoresponsive chitosan–cellulose hyaluronic acid/β-glycerophosphate (CS-HEC-HA/GP) hydrogel, which was liquid below 25 °C but could rapidly transform into a hydrogel at 37 °C. Moreover, this CS-HEC-HA/GP hydrogel loaded with human umbilical cord mesenchymal stem cells (hUC-MSCs) enhanced the retention and survival of encapsulated hUC-MSCs, promoted neurogenesis, and inhibited cell apoptosis, thereby accelerating brain structural remodeling and neurological functional recovery in TBI rats [69]. Ferulic acid (FA)-loaded injectable thermoresponsive chitosan/gelatin/β-glycerophosphate (C/G/GP) hydrogel formed a gel after about 15 min at room temperature, but only 135 s at body temperature, which ensured the in situ gelation of the C/G/GP hydrogels and effectively improved the therapeutic effect of FA on TBI [70]. Hsieh et al. synthesized two thermoresponsive water-based biodegradable polyurethane hydrogels (PU1 and PU2) loaded with neural stem cells (NSCs) through 3D bioprinting technology, which could form hydrogels at 37 °C and rescued the neural function of adult zebrafish with TBI [71]. In addition, an injectable, post-trauma ROS-responsive hydrogel with embedded curcumin (Cur) could respond to ROS stimulation in the microenvironment and reduce the oxidative stress level in the TBI lesion to decrease brain edema and promote nerve regeneration after TBI [72]. Remotely activatable ECM-mimetic chitosan/collagen-based hydrogel incorporated with mesenchymal stem cell (MSC)-membrane-coated black phosphorus (BP) exerted mild photothermal effects at the implantation site under NIR illumination, which could accelerate the healing of cranial defects [73]. Moreover, an ECM-mimetic neuroprotective sulfo-functionalized peptide hydrogel could autodegrade to generate the neuroprotective hexapeptide in the presence of MMP9 enzyme, and help repair the injured neurons in TBI lesions [74]. These studies highlighted the potential of stimuli-responsive hydrogels for TBI applications due to the functional recovery or precise brain delivery. However, new approaches and further efforts are necessary to improve the biocompatibility and reduce toxicity of stimuli-responsive hydrogels, and expand their applications in preclinical studies, thus laying the foundation for their clinical applications in the future.

## 4. Therapeutic Mechanisms of Stimuli-Responsive Hydrogels in TBI

### 4.1. Pro-Neurogenesis

Neurogenesis is a multi-step process referring to the regeneration or repair of neural tissue, which plays vital roles in learning and memory function, recovery after neuronal injury, and neural plasticity [75]. Injectable hydrogels can encapsulate various cells, drugs, or cytokines, secrete a variety of growth factors, and improve the microenvironment in the lesions to promote neurogenesis and improve neurological function after TBI.

Ma et al. found that a sodium alginate/collagen/stromal cell-derived factor-1 hydrogel loaded with bone marrow-derived mesenchymal stem cells (BMSCs/SA/Col/SDF-1) promoted neurological function recovery after TBI by enhancing hippocampal neurogenesis, as evidenced by higher EdU^+^/NeuN^+^ cells and the expression of neurotrophic factors [76]. A dual-enzymatically crosslinked injectable gelatin hydrogel loaded with BMSC improved neurological function recovery of TBI rats and promoted the proliferation of endogenous neural cells by inhibiting apoptosis and secreting neurotrophic factors [77]. Zheng et al. developed an injectable hydrogel containing polydopamine/stromal cell-derived factor-1α (PDA/SDF-1α) nanoparticles using imidazole-modified gelatin methacrylate (GelMA-imid). The results showed that the GelMA-imid/SDF-1α hydrogel loaded with human amniotic mesenchymal stromal cells (hAMSCs) facilitated the regeneration of endogenous neural cells with high expression of neuronal nuclei (NeuN), neuron-specific enolase (NSE), and brain-derived neurotrophic factor (BDNF), which had great potential for the recovery from TBI [78]. Besides, Shi et al. inoculated hUC-MSCs and activated astrocytes into a specific self-assembled peptide hydrogel RADA16-BDNF scaffold (R-B-SPH scaffold). Further, the R-B-SPH scaffold could promote neuronal regeneration and neural network reconstruction after TBI [79]. After co-culture, the expression of transcription factor SOX2, neurotrophic factor BDNF, and its receptor TRKB were upregulated. In addition, SDF-1 and hypoxia-inducible factor (HIF-1) were highly expressed at the injured site, which promoted nerve regeneration and differentiation in the injured site. Furthermore, the implantation of agarose hydrogel, which can release the Fas ligand (FasL) to induce apoptosis of cytotoxic CD8^+^ T cells in the lesion, significantly increased the expression of the nerve growth factors (NGF), BDNF and insulin-like growth factor (IGF), and promoted neural regeneration by activating the PI3K–AKT pathway in a rat model of TBI [80]. Thus, these data demonstrated that hydrogels could promote neurogenesis for repairing brain injury through encapsulated stem cells or proteins.

### 4.2. Anti-Inflammation

Inflammation is a dynamic response of the immune system to external stimuli or infection. However, when it is uncontrolled, cytokine storms (IL-1β, TNF-α, IL-6, etc.) occur, leading to the occurrence of various inflammatory diseases, including neurological diseases [81]. TBI can induce microglial activation and microglia polarization (M1 and M2) in the early stage, followed by macrophage infiltration. At the same time, pro-inflammatory factors induced by M1 microglia, such as interleukins (IL-1, IL-12, IL-18), gamma-interferon (IFN-γ), and tumor necrosis factor-α (TNF-α), and anti-inflammatory factors released by M2 microglia, such as interleukins (IL-4, IL-10, IL-13), α-interferon (IFN-α), etc., have competitive effects that can respectively promote or inhibit neuroinflammatory responses [5]. Therefore, neuroinflammation has a double-edged role in the functional remodeling of TBI. On the one hand, the inflammatory response can promote the response to external stimuli and promote the removal of cell debris, which is beneficial to the outcome after TBI; on the other hand, persistent chronic inflammation further accelerates neuronal death and progressive neurodegeneration [82]. Therefore, anti-inflammatory therapy is one of the strategies for the treatment of TBI, but attention should be paid to the time window of treatment and the course of the disease. Hydrogels can controllably release stem cells or anti-inflammatory drugs, improve drug availability, and have broad application prospects in the treatment of TBI [10].

Maclean et al. developed a multifunctional self-assembled peptide hydrogel, Fmoc-DIKVA, and encapsulated the anti-inflammatory agent fucoidan to treat TBI, which reduced primary glial scarring, significantly changed astrocyte morphology, and attenuated “reactive” astrocytosis at the injured site after 7 days after treatment for TBI [83]. Besides, the localized delivery of dexamethasone at a low dose via hyaluronic acid/PEG-bis-(acryloyloxyacetate) (PEG-bis-AA) composite hydrogels significantly reduced microglial activation/macrophage infiltration-mediated neuroinflammation and cell apoptosis, and promoted the recovery of motor function in TBI [84]. In addition, the transplantation of human neural stem/progenitor cells (hNS/PCs) and human adipose-derived stromal/stem cells (hADSCs)-loaded PuraMatrix (PM) hydrogels obviously inhibited the expression of TNF-α, IL-1α, and IL-6, and reduced reactive gliosis at the injury site, which is expected to reduce neuroinflammation and promote tissue remodeling and repair after TBI [85]. Moreover, SA/Col/SDF-1 neural scaffold loaded with BMSCs promoted neurological function recovery by mitigating neuroinflammation after TBI, as evidenced by less glial fibrillary acidic protein (GFAP)-positive cells and lower secretion of IL-1β and IL-6 [76]. In addition, the tyramine-modified hyaluronic acid hydrogels (HT) encapsulated with BMSC and NGF facilitated the proliferation of endogenous neural cells, probably by neurotrophic factor release and neuroinflammation regulation, and consequently improved the neurological function recovery of TBI [86]. Furthermore, the implantation of brain-derived ECM hydrogel ameliorated TBI-induced gliosis and microglial pro-inflammatory responses, thereby providing a favorable microenvironment for tissue repair and neurological recovery [87]. Taken together, injectable hydrogels hold promise as a potential therapeutic implant or cell/drug delivery vehicle for TBI tissue repair via anti-inflammatory treatment. However, there is an urgent need to further explore the complexity of the immune response after TBI and the optimal timing of treatment.

### 4.3. Anti-Apoptosis

Apoptosis plays an important role in neurological diseases, and inhibiting apoptosis of neural cells at the injury site is beneficial for the reconstruction of neural function [88]. The main genes involved in cell death are apoptosis inhibitors (Bcl-2) and apoptosis promoters (Bax). Studies have shown that injectable hydrogels can reduce neuronal apoptosis and promote neuronal cell survival. For example, self-assembling peptide nanofibrous hydrogel reduced acute brain injury by lowering the number of apoptotic cells, reducing the inflammatory response, as well as promoting cell survival, which may be a promising strategy in nerve repair after TBI [89]. Besides, dual-enzymatically crosslinked gelatin hydrogel significantly attenuated neuronal apoptosis, ameliorated inflammation, and facilitated the survival and proliferation of endogenous neural cells in TBI mice [90]. In addition, the transplantation of CS-HEC (chitosan, hydroxyethyl cellulose)-HA/GP thermoresponsive hydrogel loaded with hUC-MSC promoted the survival/proliferation of endogenous neurons by suppressing cell apoptosis in the brain of TBI mice [69]. Liu et al. added HA into chitosan-based self-healing hydrogels (CH). After injection into the TBI injury site, the expression of C-C-sequence chemokine ligand 2 (CCL2), toll-like receptor 2 (TLR2), IL-1β, and caspase-3 was significantly downregulated, indicating that CH hydrogel inhibited the inflammatory environment and cell apoptosis [91]. Collectively, the cell/drug-loaded hydrogels could reduce neuronal apoptosis and exert favorable effects on the neural network construction of TBI.

### 4.4. Anti-Oxidation

Hypoxia at the injured site after TBI can cause glutamate excitotoxicity and calcium influx, leading to the production of free radicals and oxidative stress, and then induce lipid peroxidation, DNA damage, and enzyme inactivation, which is not conducive to cell survival [92]. Therefore, functionalized hydrogels with the capacity for free radical scavenging or oxidative stress suppression is beneficial for TBI recovery and functional reconstruction.

For instance, Zhang et al. prepared an injectable hydrogel (HT/HGA hydrogel) by using antioxidant gallic acid-grafted hyaluronic acid (HGA) combined with hyaluronic acid–tyramine (HT) polymer. They found that the in situ injection of HT/HGA hydrogel with reactive oxygen species-scavenging activity significantly promoted the recovery of motor, learning, and memory functions of TBI mice by suppressing oxidative stress via the activation the of Nrf2/HO-1 pathway [93]. In addition, Qian et al. designed an injectable TM/PC hydrogel loaded with Cur that responded to a post-traumatic microenvironment (such as MMPs enzymes and ROS). This hydrogel could effectively respond to the TBI environment, and then depleted ROS and reduced brain edema to promote the regeneration and recovery of neurons [72]. Moreover, the transplantation of an injectable thermosensitive chitosan/gelatin/β-glycerol phosphate (C/G/GP) hydrogel with the controlled release of FA (a phenolic antioxidant) significantly reduced the expression of ROS, inflammation, and apoptosis-related markers at the injury site of TBI, and then ameliorated the secondary brain injury by reducing the level of oxidative stress at the injury site [70]. Furthermore, an oxi-methylcellulose-adipic acid dihydrazide (oxi-MC-ADH) hydrogel prepared as a vitamin C carrier could quickly diffuse ROS from the injured site after an acute TBI, and the subsequent 3-day sustained vitamin C release could scavenge the ROS to avoid consecutive neuronal degeneration that might lead to more serious sequelae [94]. Above all, antioxidant hydrogels show great potential in reducing secondary TBI, indicating the great value of this novel biomaterial in remodeling brain structure and function.

### 4.5. Pro-Angiogenesis

Vascular damage is a general pathological process in many neurodegenerative diseases as well as TBI. Angiogenesis, a highly regulated process involving the activation, migration and proliferation of vascular endothelial cells (ECs) and the formation of new blood vessels, is fundamental to brain development and repair, which contributes to the reconstruction of microenvironment for neural regeneration in the lesion area [95]. Hydrogels with angiogenic properties are ideal materials to promote angiogenesis, that is, to provide vascular supply to ischemic areas and transplanted cells. Lu et al. found that the HA-KLT (KLTWQELYQLKYKGI, a VEGF mimetic peptide,) hydrogel developed via modifying HA with a vascular endothelial growth factor (VEGF) mimetic peptide of KLT significantly improved the expression of endoglin/CD105 and enhanced the formation of blood vessels in brain lesions [96]. Besides, self-assembling peptide-based hydrogel (SAPHs) created a regenerative microenvironment for neovascularization at the injury site of TBI, which activated angiogenesis by inducing ECs adhesion and upregulating VEGF-R2, which in turn promotes axonal growth and neuronal survival [97]. In addition, a functionalized self-assembling nanopeptide hydrogel could induce angiogenesis with the expression of enhanced green fluorescent protein (EGFP) and α-smooth muscle actin (α-SMA) by ECs and pericytes, to help the reconstruction of damaged neural tissue and promote functional recovery from TBI [98]. Similarly, the combined therapy of self-assembling peptide nanofiber hydrogel (dual-functionalized with VEGF- and BDNF-mimetic peptides) and chitosan conduits synergistically contributed to the healing of injured nerves via enhancing angiogenesis and neurogenesis [99]. Thus, strategies to promote angiogenesis and neurogenesis by functionalized hydrogels have potential applications in neural tissue engineering, providing new ideas for TBI therapy.

## 5. Conclusions and Prospects

**Figure 5:**
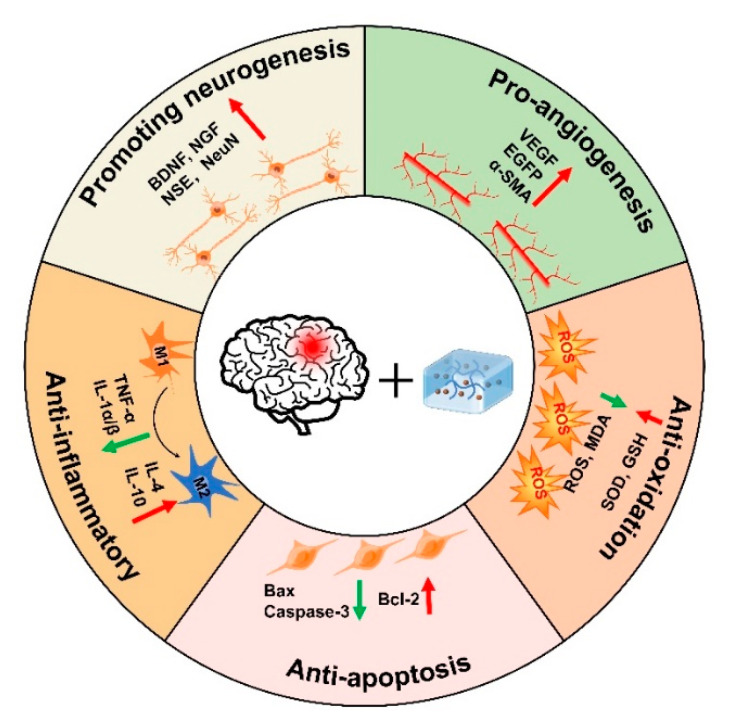
Therapeutic mechanisms of injectable hydrogels in TBI.

Since the microenvironment of TBI is not conducive to cell survival, and the BBB hinders drug transport, there is an urgent need to explore novel strategies to promote nerve regeneration. In this paper, we briefly described the characteristics and gelation mechanisms of thermoresponsive hydrogels, photoresponsive hydrogels, magnetic-responsive hydrogels, electroresponsive hydrogels, and bioresponsive hydrogels, and enumerated the applications and therapeutic mechanisms of stimuli-responsive hydrogels in TBI, including pro-neurogenesis, anti-inflammatory, anti-apoptosis, anti-oxidation, and pro-angiogenesis (Figure 5, Table 1). Although great progress has been made in current research on stimuli-responsive hydrogels, stimuli-responsive hydrogels still have some limitations, such as thermal denaturation, UV carcinogenesis, low mechanical strength, and material toxicity. Furthermore, the high swelling properties of hydrogels may increase local tissue pressure, which not only deteriorate the mechanical property of the hydrogels but also can bring about secondary brain injury after TBI. Therefore, it is necessary to improve injection conditions to avoid additional damage to the brain, such as using syringes equipped with smaller needles to deliver small volumes at more controlled rates. In addition, the lesion volume, the location of injection, and the unique swelling properties of hydrogels formulation may all influence the acceptable range of injection volumes in the TBI lesion. Therefore, it is crucial for researchers to continue developing improved hydrogel fabrication strategies to better control the swelling of hydrogels. Taken together, future studies should preferentially focus on improving the biocompatibility of stimuli-responsive hydrogels with the body, especially deepening the study of natural materials. In addition, the optimal time for hydrogel treatment should be further explored.

## Figures and Tables

**Table 1:** Therapeutic mechanisms of injectable hydrogels in TBI.

Molecular Mechanisms	Hydrogels	Components	Loaded Materials	Models	Refs
Pro-neurogenesis	SA/Col hydrogel	SA, Col	BMSCs, SDF-1	SD Rats	[76]
	Gelatin hydrogel	Gelatin, GOX, HRP	BMSCs	SD Rats	[77]
	GelMA-IMID hydrogel	Gelatin, MA, IMID	hAMSCs, PDA@SDF-1α	SD Rats	[78]
	RADA16-BDNF peptide scaffolds	RADA16, RGI	CXCR4, hUC-MSCs, activated astrocytes	SD Rats	[79]
	Agarose hydrogel	Agarose	FasL	SD Rats	[80]
Anti-inflammatory	SA/Col hydrogel	SA, Col	BMSCs, SDF-1	SD Rats	[76]
	Fmoc-DIKVAV hydrogel	Fmoc	fucoidan	C57 BL/6 mice	[83]
	PEG-bis-AA/HA-DXM hydrogel	PEG, AA, HA	DXM	SD Rats	[84]
	BD™PuraMatrix™ hydrogel	PuraMatrix	hNS/PCs, hADSCs	SD Rats	[85]
	HA gelatin	HA, GOX, HRP	NGF, BMSCs	C57BL/6	[86]
	ECM hydrogel	ECM	/	C57 BL/6 mice	[87]
Anti-apoptosis	CS-HEC-HA/GP hydrogel	CS, HEC, HA, GP	hUC-MSCs	SD Rats	[69]
	GH hydrogel	gelatin, HRP, ChOx	BMSCs	C57BL/6 mice	[90]
	CH hydrogel	HA, CS	/	SD Rats, Zebrafish	[91]
Anti-oxidation	HT/HGA hydrogel	HA, Tyr	GA	C57BL/6 mice	[93]
	oxi-MC-ADH-VC hydrogel	MC	Vitamin C	SD Rats	[94]
	TM/PC hydrogel	TM, PPS120	Cur	ICR mice	[72]
	C/G/GP hydrogel	CS, gelatin, GP	FA	N2a cells	[70]
Pro-angiogenesis	HA-KLT hydrogel	HA	VEGF mimetic peptide KLT	SD Rats	[96]
	SAPH	Slanc	/	SD Rats	[97]
	SAPH	RADA16-SVVYGLR	/	Zebrafish	[98]
	SAPH	RAD	KLT, RGI	SD Rats	[99]

## Data Availability

Not applicable.

## Abbreviations

TBI, traumatic brain injury; BBB, blood–brain barrier; GCS, Glasgow Coma Score; pNIPAAm, poly (*N*-isopropylacrylamide); PEO-PPO-PEO, poly (ethylene oxide)-poly (propylene oxide)-poly (ethylene oxide); HA, hyaluronic acid; PEG, polyethylene glycol; LCST, lower critical solution temperature; UCST, upper critical solution temperature; BPNs, black phosphorus nanosheets; PRP, platelette-rich plasma; RA, rheumatoid arthritis; PPS-b-PDMA-b-PNIPAAM, poly [(propylenesulfide)-block-(*N*,*N*-dimethylacrylamide)-block-(*N*-isopropylacrylamide)]; UV, ultraviolet; NIR, near infrared; BMH, bio-inspired MnO_2_ hybrid; UCNPs, upconverting nanoparticles; AMF, alternating magnetic field; PPy, polypyrrole; PANI, polyaniline; PEDOT, poly(3,4-ethylenedioxythiophene); PPP, PLGA-PEI-MPEG; ACPP, activated cell-penetrating peptides; ET, etanercept; MMPs, matrix metalloproteinases; spinal cord injury, SCI; PKA, protein kinase A; H-IgG, human immunoglobulin G; BP, black phosphorus; ECM, extracellular matrix; HSPs, heat shock proteins; CS-HSE-HA/GP, chitosan hydroxyethyl cellulose hyaluronic acid/β-glycerophosphate; FA, Ferulic acid; C/G/GP, chitosan/gelatin/β-glycerophosphate; PU, polyurethane; NSCs, neural stem cells; Cur, curcumin; MSC, mesenchymal stem cell; SA, sodium alginate; Col, collagen; SDF-1, stromalcell-derivedfactor-1; BMSCs, bone marrow-derived mesenchymal stem cells; PDA, polydopamine; SDF-1α, stromal-cell derived factor-1α; hAMSCs, human amniotic mesenchymal stromal cells; GelMA-imid, imidazole groups-modified gelatin methacrylate; NeuN, neuronal nuclei; NSE, neuron specific enolase; BDNF, brain derived neurotrophic factor; hUC-MSCs, human umbilical cord mesenchymal stem cells; R-B-SPH, self-assembled peptide hydrogel RADA16-BDNF; HIF-1, hypoxia-inducible factor; FasL, Fas ligand; NGF, nerve growth factor; IGF, insulin-like growth factor; TNF-α, tumor necrosis factor-α; IL, interleukin; IFN-γ, gamma-interferon; IFN-α, α-interferon; PEG-bis-AA, PEG-bis-(acryloyloxyacetate); hADSCs, human neural stem/progenitor cells; PM, PuraMatrix; GFAP, glial fibrillary acidic protein; HT, tyramine-modified hyaluronic acid hydrogels; CCL2, C-C-sequence chemokine ligand 2; TLR2, Toll-like receptor 2; CH, chitosan-based self-healing hydrogels; MDA, malondialdehyde; GSH, glutathione; oxi-MC-ADH, oxi-methylcellulose-adipic acid dihydrazide; ECs, endothelial cells; CS-HEC, chitosan, hydroxyethyl cellulose; KLT, KLTWQELYQLKYKGI; VEGF, vascular endothelial growth factor; SAPHs, self-assembling peptide-based hydrogel; EGFP, enhanced green fluorescent protein; αSMA, α-smooth muscle actin.
